# Signet Ring Cell Carcinoma at the Ampulla of Vater: A Very Rare Diagnosis

**DOI:** 10.7759/cureus.30403

**Published:** 2022-10-17

**Authors:** Pratibha P Dawande, Faizan Akhtar, Rashmi S Wankhade, Nandkishor J Bankar

**Affiliations:** 1 Pathology, Datta Meghe Medical College, Datta Meghe Institute of Medical Sciences, Wardha, IND; 2 Microbiology, Jawaharlal Nehru Medical College, Datta Meghe Institute of Medical Sciences, Wardha, IND

**Keywords:** rare case report, adenocarcinoma, pancreas, ampulla of vater, signet ring cell type carcinoma

## Abstract

Signet ring cell carcinoma (SRCC) is a rare lesion in the gastrointestinal tract. Further, the condition is very uncommon at the ampulla of Vater. A majority of the reported cases are typical, gland-forming adenocarcinomas. In our case, a patient aged 59 years, was diagnosed as a case of peri-ampullary carcinoma based on physical exam findings and imaging. Ultrasonography (USG) abdomen and magnetic resonance cholangiopancreatography (MRCP) revealed an enlarged common bile duct (CBD) and there was a presence of stricture at the terminal CBD. Endoscopic retrograde cholangiopancreatography (ERCP) showed growth at the ampulla of Vater. An endoscopic ultrasound guided needle core biopsy was obtained. Histopathological examination revealed the case as SRCC at the ampulla of Vater. We present this as an uncommon case of SRCC at the ampulla of Vater.

## Introduction

The ampulla of Vater, which is distal to the point where the distal common bile duct (CBD) and the pancreatic duct (PD) split, is the source of ampullary tumors (ATs). Less than 0.5% of all gastrointestinal neoplasms are these uncommon tumors [1]. Because of advancements in diagnostic endoscopic and radiographic techniques, their occurrence has increased in recent years [2]. More than 95% are premalignant (like adenomas) or malignant (like adenocarcinomas), however, some are benign (like lipomas). Adenomas have a similar pattern to colorectal adenocarcinomas in the adenoma-to-carcinoma transition [3].

Ampulla of Vater is an interesting area in histopathology as there is a confluence of the pancreatic, biliary, and intestinal epithelium. Of the four tumors (pancreas, bile duct, ampulla, and periampullary duodenum), carcinoma of the ampulla of Vater is the second most prevalent, after carcinoma of the head of the pancreas, and accounts for around 10% of all periampullary tumors. The incidence of true ampullary carcinomas is relatively low and generally shows a better prognosis than peri-ATs [4]. The stomach is where signet ring cell carcinoma (SRCC) is most frequently diagnosed among the organs in the gastrointestinal tract [5]. It is extremely rare to find this condition at the ampulla of Vater [1]. In this study, we present a case report of SRCC discovered in histopathology in our institution.

## Case presentation

We present a case of a 59-year-old male who arrived at the hospital complaining of generalized weakness, yellow discoloration of the eyes, vomiting, and loss of appetite. Serum bilirubin levels were significantly elevated. Carcinoembryonic antigen (CEA) level was 739 ng/mL, and carbohydrate antigen (CA) 19-9 level was 102 units/mL. Ultrasound abdomen and magnetic resonance cholangiopancreatography (MRCP) revealed dilated CBD and stricture at the terminal CBD. Endoscopic retrograde cholangiopancreatography (ERCP) revealed growth at the ampulla of Vater. A provisional diagnosis of obstructive jaundice with periampullary carcinoma was given and a biopsy was taken from the lesion and sent for histopathological examination. On histopathological examination, the case was diagnosed as SRCC, and subsequently, the patient underwent Whipple’s procedure. The resected specimen was received for histopathological examination in our surgical pathology section.

Gross findings

The specimen consisted of the second and third parts of the duodenum together measuring 30 cm. The gall bladder was 10 cm x 3 cm x 0.5 cm in size, and the CBD was 3 cm in length. The head of the pancreas measured 5.5 cm x 3 cm x 3 cm.

**Figure 1:**
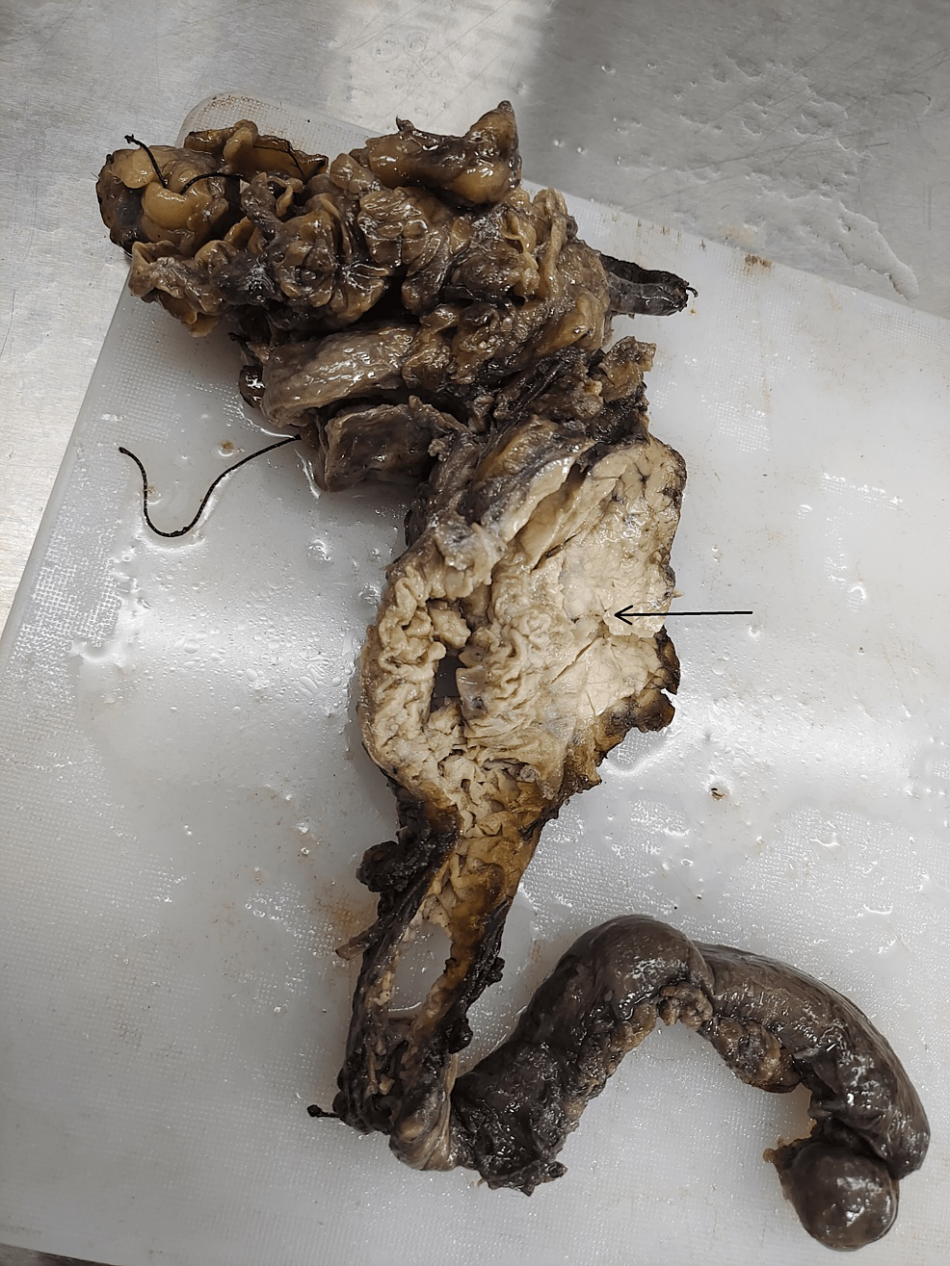
Cut section showing growth at the ampulla of Vater. Black arrow showing growth at the ampulla of Vater.

On the cut section, growth was identified at the ampulla of Vater measuring 2.5 cm x 2 cm x 1 cm, grayish white in color with areas of hemorrhage. The cut section of the gall bladder revealed velvety mucosa, and the head of the pancreas showed yellowish-to-brown areas. No lymph nodes were found. Sections were taken from ten representative areas and studied histologically (Figure 1).

Microscopic findings

Sections studied from the ampullary growth revealed mucosa lined by columnar epithelium infiltrated by diffuse sheets of signet ring cells. These cells had vacuolated cytoplasm and hyperchromatic pleomorphic eccentric nuclei. Areas of mucin pools were seen. A dense inflammatory infiltrate was seen in lamina propria, submucosa, and muscularis propria. The serosa showed inflammatory cells and congested blood vessels. Sections studied from both proximal and distal margins were free from tumor invasion. Sections studied from the duodenum, gall bladder, pancreas, mesentery, and CBD were free from tumor invasion.

Impression

**Figure 2:**
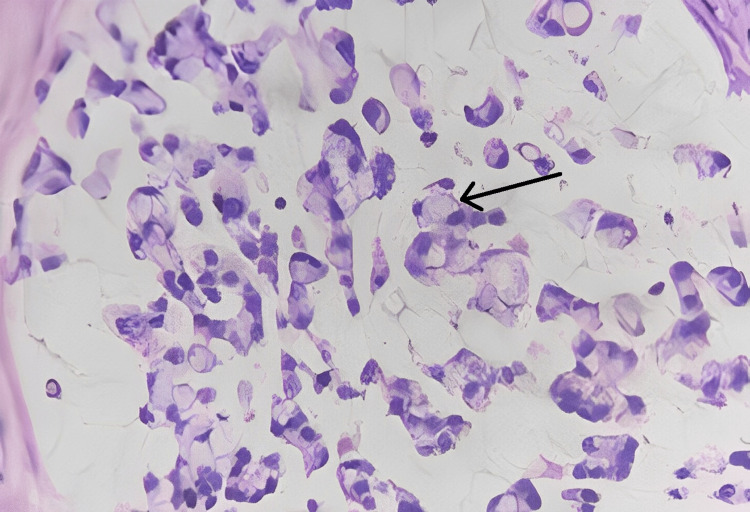
Histopathological photomicrograph showing characteristic signet ring cells with mucin (40x). Black arrow showing characteristic signet ring cells with mucin

**Figure 3:**
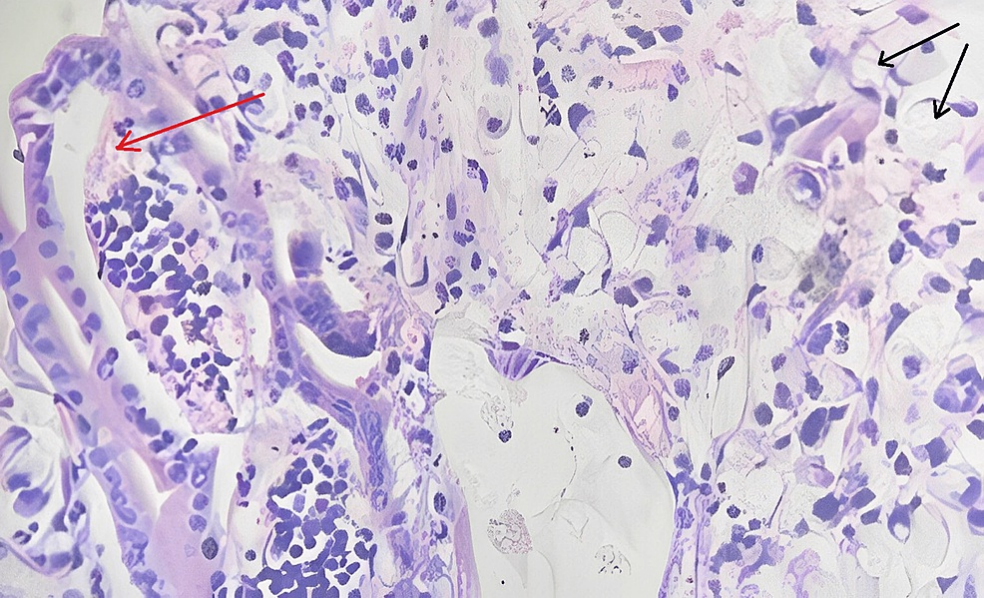
Histopathological photomicrograph showing mucosal glands with signet ring cells (40x). Red arrow shows mucosal glands and black arrow shows signet ring cells

**Figure 4:**
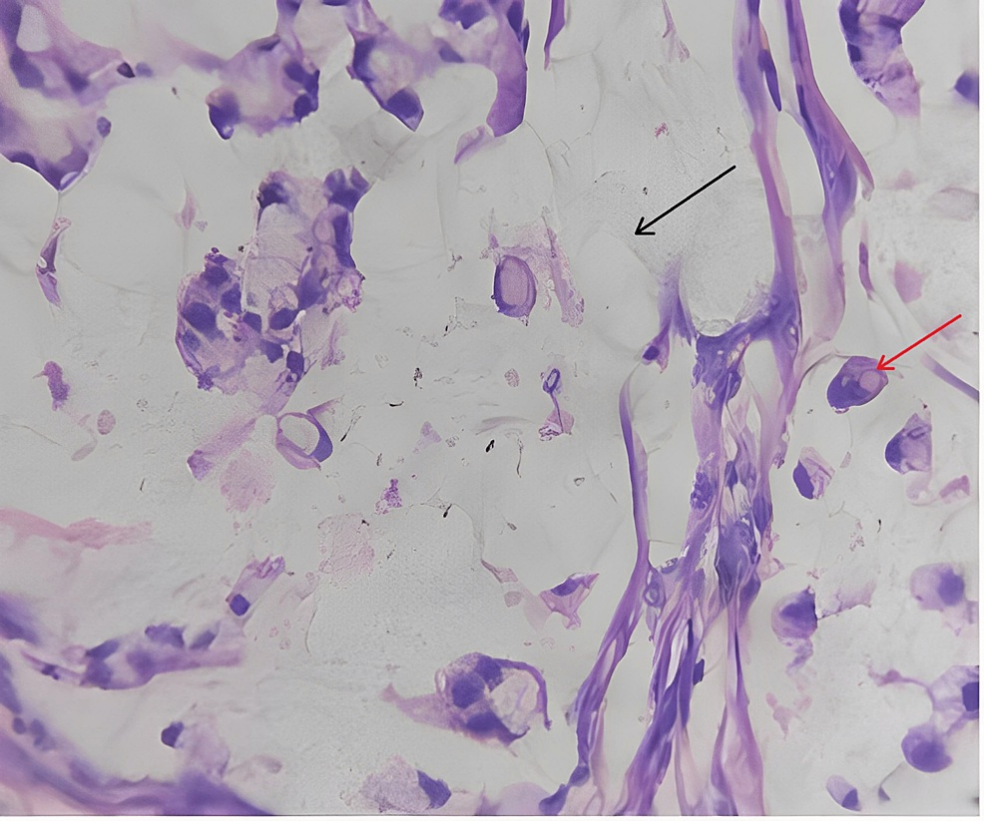
Histopathological photomicrograph showing few signet ring cells with abundant mucin (40x). Black arrow shows abundant mucin and red arrow shows signet ring cells

Signet ring cell adenocarcinoma of the ampulla of Vater (TisN0M0) Stage 0 (Figures 2-4).

## Discussion

Signet ring cell carcinoma, a variant of adenocarcinoma is identified by the presence of signet ring cells. Mucin is abundant in the cytoplasm of signet ring cells, and these cells are positive for periodic acid-Schiff-diastase (PAS-D) and mucin stains. The endoscopic biopsy results may not always be evident with early-stage tumors because the proper location of the lesion is not accessed by the endoscope [6]. 

Regarding the pathogenesis of SRCC, little is known. Histopathological examination of this neoplasm reveals signet ring cells. Fukui (2014) has offered a thorough explanation of the mechanism that results in the creation of signet ring cells, which includes the ErbB2 (Erb-B2 Receptor Tyrosine Kinase 2) /ErbB3 (HER3 human epidermal growth factor receptor 3) complex, phosphatidylinositol 3-kinase (PI3K) activation, and enhanced mucin secretion [7]. In our study, we have not done these as it was not included in the guidelines. Ampullary SRCC may be classified further into four types, i.e. intestinal, gastric, pancreato-biliary, and mixed type [8-9]. Immunohistochemistry serves as the basis for this classification. The overall median survival is 25 months, with a range of 6-132 months [10]. The main prognostic parameter appears to be lymph node involvement rather than the locational subtypes mentioned above [9, 11].

Compared to other ampullary adenocarcinomas, Kinslow et al. observed that SRCC appears to be present at a later stage and has a higher potential for bone metastasis [12]. They found a 17-month median survival time, which was worse than other ampullary adenocarcinomas. Additionally, some of the poor prognostic factors of SRCC are the older age of the patient, a later stage of diagnosis, and the fact that the condition does not enable cancer-directed surgery.

Blackman and Nash (1985) reported two anaplastic carcinomas with few signet ring cells [13]. Siefert et al. (1992) reported one case of SRCC out of 35 carcinomas of the ampulla of Vater [14]. The most prevalent histological subtypes of 170 ampullary adenocarcinomas, according to a multicenter analysis, were intestinal, pancreato-biliary, and poorly differentiated, in that order. Out of the 170 cases of ampullary adenocarcinomas, only one (0.6%) was diagnosed as SRCC [15]. Several cases of ampullary adenocarcinomas have been reported, however, SRCC at the ampulla has been rarely discussed. Hara et al. (2002) reported the condition in a 68-year-old male patient with postprandial abdominal pain and nausea [11]. Ultrasound of the abdomen similarly revealed a dilated CBD. Biopsy taken revealed SRCC of the ampulla of Vater like our case, although the resected specimen in their case revealed lymphatic and vascular invasion too. Souaf et al. (2014) reported an instance of the same condition with duodenal invasion [16]. The lymph nodes were not involved and there was no evidence of any distant metastasis. Ishibashi et al. (2009) reported a case of SRCC, which revealed the involvement of the duodenum as well as the pancreas by the tumor [17]. Akatsu et al. (2007) identified 14 instances with a median age of 57 years. These patients were around 15 years older than the average age of patients with signet ring cell tumors found in the stomach [18]. Acharya et al. (2013) presented 30 previously diagnosed cases between the ages of 32 years and 83 years [19]. An advanced disease (T4) was diagnosed in one of the patients.

Eriguchi et al. (2003) discussed a case of an 83 years old man who had complained of overall weariness, fever, and obstructive jaundice [20]. Gastroduodenal fibroscopy had identified malignancy at the ampulla of Vater. Following a biopsy, gastroduodenal SRCC was diagnosed. Ramia et al. (2004) presented a case of a 67-year-old woman who was diagnosed on histopathology as SRCC of the ampulla of Vater T2N0M0 [21]. Fornelli et al. (2019) reported a case with a tumor having elements of neuroendocrine differentiation. On phenotype, it was identified as the intestinal variant [22]. Ikeda et al. (2020) presented a case of a 74-year-old woman who had epigastric pain and was later diagnosed with cholangitis. Her liver function tests were deranged. CT scans revealed an enhanced area in the peri-ampullary region with marked dilatation of the CBD. A total of 13 biopsies were required to diagnose the case as SRCC [23].

## Conclusions

In conclusion, we reported a case of SRCC of the ampulla of Vater, which is an exceedingly rare condition. On clinical examination and imaging investigations, a diagnosis of peri-ampullary carcinoma was given. On histopathological examination, a diagnosis was confirmed as Stage 0 (TisN0M0) signet ring cell adenocarcinoma. Although there are few studies or case reports published on this entity, more studies should be done on the pathogenesis and to understand the outcomes and prognosis of various stages of the tumor.
